# Prehabilitation Consultation on Self-Care and Physical Exercise in Patients Diagnosed with Abdominopelvic Cancer: Protocol of the Study

**DOI:** 10.3390/healthcare12141423

**Published:** 2024-07-16

**Authors:** María Pilar Suárez-Alcázar, Eladio J. Collado-Boira, Paula Recacha-Ponce, Pablo Salas-Medina, M. Elena García-Roca, Carlos Hernando, María Muriach, Pablo Baliño, Raquel Flores-Buils, María Luisa Martínez Latorre, Nerea Sales-Balaguer, A. Folch-Ayora

**Affiliations:** 1 Nursing Department, University of Jaime I, Av. Vicente Sos Baynat s/n, 12071 Castellón de la Plana, Castellón, Spain; malcazar@uji.es (M.P.S.-A.); colladoe@uji.es (E.J.C.-B.); recacha@uji.es (P.R.-P.); garciroc@uji.es (M.E.G.-R.); afolch@uji.es (A.F.-A.); 2 Department of Education and Specific Didactics, University of Jaume I, Av. Vicente Sos Baynat s/n, 12071 Castellón de la Plana, Castellón, Spain; hernando@uji.es; 3 Medicine Department, University of Jaime I, Av. Vicente Sos Baynat s/n, 12071 Castellón de la Plana, Castellón, Spain; muriach@uji.es (M.M.); balino@uji.es (P.B.); 4 Department of Developmental, Educational, Social and Methodology Psychology, University of Jaime I, Av. Vicente Sos Baynat s/n, 12071 Castellón de la Plana, Castellón, Spain; flores@uji.es; 5 Asociación Española Contra el Cáncer, Passeig de Ribalta n° 25–27, 12001 Castellón de la Plana, Castellón, Spain; luisa.martinez@contraelcancer.es; 6 PhD Programme in Biomedical Sciences and Health, University of Jaime I, Av. Vicente Sos Baynat s/n, 12071 Castellón de la Plana, Castellón, Spain; nereasalesnutricion@gmail.com

**Keywords:** prehabilitation, abdominopelvic cancer, physical exercise, self-care

## Abstract

Background: Introduction: Prehabilitation in the field of oncology has been defined as “the process in the continuum of care that occurs between diagnosis and the start of treatment involving physical and psychological measures that determine the patient’s baseline functional status.” Aim: To determine the effectiveness of a Prehabilitation consultation on self-care and targeted physical exercise for patients diagnosed with abdominopelvic cancer. Design: An observational study that will evaluate the pre-post efficacy of an ad-hoc designed Prehabilitation intervention. The study population consists of patients diagnosed with colon or gynecological cancer with a surgical indication as part of their therapeutic plan from the General Surgery Services. It is configured around four key interventions: (a) health education and self-care, (b) specific nutritional counseling, (c) initial psychological assessment, and (d) directed physical exercise intervention. Health education, self-care interventions, and physical exercise will be carried out weekly from diagnosis to the scheduled surgery day. Results: Aspects such as self-care capacity or agency, perioperative anxiety, aerobic capacity, strength and flexibility, postoperative complications, and recovery time to adjuvant treatment will be measured using tools such as Appraisal of self-care agency scale (ASA), State Trait Anxiety Inventory (STAI), walking test, sit and Reach, Hand Grip or Squad Jump. Conclusion: Utilizing validated tools for analyzing selected variables will contribute to refining and expanding care guidelines, ultimately enhancing support for both patients and their caregivers.

## 1. Introduction

Prehabilitation in the field of oncology has been defined as the process within the continuum of care that occurs between diagnosis and the onset of acute treatment, involving physical and psychological measures that determine the patient’s baseline functional status [1,2,3,4,5,6].

This type of intervention is distinct from medical optimization of comorbid conditions addressed through Enhanced Recovery After Surgery (ERAS) protocols, which focus on intensified postoperative recovery procedures [7]. Currently widely implemented in the surgical field, ERAS protocols originally focused on preoperative interventions within the 24 h leading up to surgery [7]. However, there is a growing interest in the development and implementation of interventions at even earlier stages of cancer treatment (immediately after diagnosis) [2,7].

Multimodal Prehabilitation entails a continuum of care from the moment of diagnosis to the initiation of treatment, providing an opportunity to support and prepare patients for the planned therapeutic regimen and to enhance their self-care agency or capacity [4]. Furthermore, it offers the opportunity to transform a waiting period characterized by a passive role until surgery into an opportunity for patient empowerment and active optimization.

These interventions have become an important strategy for improving the physiological and psychosocial health of patients before cancer treatment and for preserving their quality of life [1,8]. Prehabilitation provides the opportunity to improve physical function [8,9,10,11,12], nutrition [8], and mental function of patients [8,10,11] and to optimize their pre-surgical state [9]. Furthermore, it has a plausible physiological effect and is consistent with the basic principles of current ERAS protocols [7,13].

Prehabilitation programs in cancer described in current literature include interventions related to the cessation of toxic habits such as smoking and alcohol consumption [1,2,6], nutritional counseling [1,2,3,6,7,8,9,10,14,15,16,17,18,19], physical exercise [1,2,3,6,7,8,9,10,14,15,16,17,18,19] and psychological support [1,2,3,6,7,8,9,10,14,15,16,17,18,19]. However, studies related to prehabilitation in abdominopelvic cancer are highly heterogeneous in terms of intervention and investigated outcomes. For example, some of the protocols described in the literature include only some of these interventions [20], and others include patients undergoing surgery alongside patients undergoing other cancer treatments [21]. Further evidence is needed to determine the optimal Prehabilitation program for these patients, and our protocol includes not only the four basic modalities of multimodal prehabilitation in patients under surgery but also the improvement of the self-care capacity of these patients.

### 1.1. Hypotheses

- Participation of patients with colon or gynecological cancer in a Prehabilitation program focusing on self-care and targeted physical exercise improves their self-care capacity or agency and decreases their preoperative anxiety levels;
- Participation of patients with colon or gynecological cancer in a Prehabilitation program focusing on self-care and targeted physical exercise improves their aerobic capacity, strength, and flexibility before surgery;
- Participation of patients with colon or gynecological cancer in a Prehabilitation program focusing on self-care and targeted physical exercise reduces the occurrence of postoperative complications and reduces the postoperative recovery time until the start of adjuvant therapy.

### 1.2. Aims

The main objective of this research is to determine the effectiveness of a Prehabilitation consultation focusing on self-care and targeted physical exercise for patients diagnosed with abdominopelvic cancer.

Additionally, the following specific objectives have been formulated:

- Establish whether there is a decrease in preoperative anxiety levels in patients with colon or gynecological cancer after their participation in the Prehabilitation program focusing on self-care and targeted physical exercise;
- Determine whether there is an improvement in aerobic capacity, strength, and flexibility in patients with colon or gynecological cancer after their participation in the Prehabilitation program focusing on self-care and targeted physical exercise;
- Determine whether there is a reduction in the occurrence of postoperative complications in patients with colon or gynecological cancer after their participation in the Prehabilitation program focusing on self-care and targeted physical exercise;
- Determine whether there is a reduction in the postoperative recovery time until the start of adjuvant therapy in patients with colon or gynecological cancer after their participation in the Prehabilitation program focusing on self-care and targeted physical exercise.

## 2. Materials and Methods

### 2.1. Sample

The study population consists of patients diagnosed with colon or gynecological cancer who have a surgical indication as part of their therapeutic plan, originating from the Surgical Services of various public hospitals within the province.

#### 2.1.1. Selection Criteria

Inclusion Criteria:

- Age over 18 years.
- Diagnosis of colon or gynecological cancer with a surgical indication as part of their therapeutic plan from the General Surgery Services of various public hospitals in the province;
- Attendance to at least 70% of physical exercise sessions;
- Anticipated participation in the program for at least 2 weeks;
- Capacity to provide informed consent;

Exclusion Criteria:

- Inability to understand provided information or insufficient knowledge of the Spanish language;
- Inability to carry out the scheduled intervention as per physician’s criteria;
- Emergency surgery due to obstruction, perforation, hemorrhage, or similar reasons during the prehabilitation period;
- Receipt of neoadjuvant or adjuvant treatment until colon or gynecological surgery.

Intervention: The responsible hospital surgeons and collaborators from various Surgery Services will facilitate contact between patients who meet the established inclusion criteria with the prehabilitation consultation at the University of Jaume I.

Physicians will provide patients with an informational sheet about the project, which they can fill out to request information about the study. This informational sheet will be collected weekly by project coordinators.

From the prehabilitation consultation, initial telephone contact will be made with patients who have requested information about the project, and they will be scheduled for an in-person meeting to provide them with information about the study, the data collection methods, and the planned procedures to ensure their anonymity and confidentiality.

Contact with study participants will always be made directly, and after the described recruitment process, written informed consent for voluntary participation will be obtained.

Participation in the research study does not involve any medical techniques or invasive procedures that could affect participants. Likewise, there is no experimental component that could jeopardize their health.

The prehabilitation program has been structured around four key interventions: (a) health education and self-care, (b) specific nutritional counseling, (c) initial psychological assessment, and (d) targeted physical exercise intervention.

#### 2.1.2. Health Education and Self-Care Interventions

The health education and self-care interventions will be conducted in one in-person session on a weekly basis, each lasting approximately 30 min. During these sessions, participants in the prehabilitation program will receive general dietary guidelines emphasizing a healthy diet, with a focus on adequate protein intake, as well as information on the source and quality of protein intake. They will also receive general guidelines regarding alcohol and tobacco consumption, along with information on the benefits of abstinence prior to surgery and the health risks associated with the consumption of these substances. Additionally, patients included in the program will receive reinforcement of postoperative instructions provided by the surgical team and information about the surgical procedure they will undergo.

These sessions will be conducted by the nurse and doctoral candidate in Biomedical and Health Sciences as the principal investigator of the project and will be supported by specialists in physical activity and sports, surgery, nutrition, and psycho-oncology who have collaborated in the development of this prehabilitation intervention program.

#### 2.1.3. Specific Nutritional Counseling

Participants in the prehabilitation program will have access to a specific nutritional counseling consultation, which can be conducted online or in person, according to the preference of the patients themselves.

#### 2.1.4. Initial Psychological Support

Participants in the prehabilitation program will have access to an initial psychological support consultation for patients with an oncological diagnosis, which can be conducted online or in person, according to the preference of the patients themselves.

#### 2.1.5. Directed Physical Exercise Intervention

Participants in the Prehabilitation program will engage in planned, supervised, and personalized exercise sessions tailored to their baseline physical condition and oncological pathology under the prescription of their therapeutic plan providers. These sessions will occur twice a week, lasting one hour each, from the time of inclusion in the study until the scheduled surgery date.

The directed physical exercise sessions will be supervised by a licensed graduate in Physical Activity and Sport Sciences, specializing in Physical Activity and Oncology, who is collaborating on the project and is a member of the Care and Health research team at Jaume I University.

The planned physical exercise sessions are structured as follows:

1. Warm-up: The aim is to prepare the body, both physically and psychologically, for the type of activity to be performed. The duration is between 5 and 10 min;
2. Main part: The planned exercise routine for that day will be executed, including explanations, demonstrations, execution, and practice of all exercises, with varied exercises suitable for different physical abilities. An exercise regimen will enhance both upper and lower body strength as well as cardiorespiratory fitness by engaging all major muscle groups through the use of body weight, resistance bands, free weights, and exercise mats. The program incorporates a combined circuit of 8–12 functional exercises, including squats, front and side lunges, sit-ups, calf raises, glute bridges, core exercises, biceps curls, shoulder presses, punches, jumping jacks, and static walking/jogging. The circuit comprises two sets of 10–12 repetitions for the strength exercises and 30 s for the aerobic exercises. Exercise volume was progressively increased by adjusting the number of repetitions, sets, and exercise complexity. A minimum rest period of 90 s was maintained between exercises. The duration is between 30 and 35 min;
3. Cool-down and relaxation: The objective is to bring the heart rate back to normal values, perform deep and prolonged stretches of the muscle groups involved throughout the session, and dedicate a few minutes to relaxation as part of the psychological intervention of the program. The duration is between 5 and 10 min.

This section comprised a combined circuit of 8–12 functional exercises, including squats, front and side lunges, sit-ups, calf raises, glute bridges, core exercises, biceps curls, shoulder presses, punches, jumping jacks, and static walking/jogging. The circuit consisted of 2 sets of 10–12 repetitions for the functional strength exercises and 30 s for the aerobic exercises. The volume was progressively increased by adjusting the number of repetitions and sets, as well as the complexity of the exercises. A minimum rest period of 90 s was maintained between exercises.

Variables: A series of primary variables related to the research topic and secondary variables of sociodemographic nature (age, marital status, presence of children, smoking status, gender, level of education, employment status) and clinical nature (cancer type, tumor markers, type of scheduled surgery, postoperative complications, length of hospital stay, ostomy, postoperative recovery time until adjuvant therapy, reinterventions, and readmissions) of the patients included in the study will be collected. The different selected primary variables and the procedures for obtaining them and/or the selected measurement tools and instruments are detailed below (Table 1)

### 2.2. Assessment

Two assessments are planned throughout the prehabilitation program for the collection of variables of interest, which will be carried out by a single standardized evaluator, the doctoral student responsible for the research project:

- Baseline Assessment: This assessment will take place after the inclusion of patients in the Prehabilitation program and before starting the directed physical exercise intervention. In this initial assessment, data will be collected on sociodemographic variables (age, marital status, level of education, employment status, presence of children, and profession), pre-surgical clinical variables (type and subtype of cancer, CA 19.9, CEA, type of scheduled surgery), physical variables (body composition, heart rate, oxygen saturation, blood pressure), physical fitness variables (aerobic capacity, strength, and flexibility), psychological variables (anxiety and depression), and self-care and health variables (self-care agency, quality of life, toxic habits, nutritional status, frailty). Additionally, patients will be fitted with an accelerometer during this assessment, which they will wear until the scheduled intervention date for lifestyle assessment. This accelerometer will be placed on the non-dominant hand and will assess the level of activity of these patients over time (from the baseline assessment to the pre-surgical assessment). This information will include the percentage of minutes per day that patients spend engaging in sedentary, vigorous, or very vigorous activities. Similarly, it will provide information on hours of nighttime rest;
- Pre-surgical Assessment: This assessment will be conducted as close as possible to the surgery date, and it will involve a re-evaluation of psychological variables (anxiety and depression), self-care and health variables (self-care agency, quality of life, toxic habits), and a reassessment of physical variables (body composition, heart rate, oxygen saturation, blood pressure), physical fitness variables (aerobic capacity, strength, and flexibility). Additionally, the accelerometer placed on patients during the baseline assessment for lifestyle assessment will be removed during this assessment.

One month after the completion of the intervention, information will be collected regarding postoperative clinical variables (postoperative complications, length of hospital stays, ostomy, postoperative recovery time until adjuvant therapy, reinterventions, and readmissions).

Finally, this study includes comparing the postoperative variables collected after the intervention with those of a control group through medical record review and a retrospective process of these same postoperative variables collected in a population with the same characteristics as the study sample, recorded during the same period as the designed intervention, but from the year prior to it.

The interventions and assessments included in the Prehabilitation program will be conducted in person at the facilities of the chair of Physical Activity and Oncology at the University of Jaume I.

Data Management: The handling of data from subjects participating in the study will comply with the provisions set forth in Spanish Law 41/2002 of November 14 on basic regulations governing patient autonomy and rights and obligations regarding information and clinical documentation, as well as Spanish Organic Law 3/2018 of December 5, on the protection of personal data and guarantee of digital rights.

A pseudonymization of participants will be conducted by assigning a unique code to each patient resulting from the combination of their data (23PREHABXXXYY), where XXX represents the subject’s initials, and YY represents the year of birth. Confidentiality of the data will be ensured, and access to the data will be restricted to members of the research team, who will be responsible for the protection of participants’ data.

Statistical analysis: The statistical analysis will be performed using IBM SPSS Statistics version 29 (IBM Corporation, Armonk, NY, USA).

A descriptive analysis of the variables will be conducted using measures of central tendency and standard deviations for quantitative variables, and frequencies and percentages for qualitative variables.

Regarding inferential analysis, the Chi-square test (categorical variables) will be employed, along with parametric and non-parametric analyses (Student’s *t*-test, ANOVA, Mann–Whitney U test, or Kruskal–Wallis H test), and Spearman or Pearson correlation analysis depending on the observed normality for quantitative variables. In all cases, a statistical significance level of 5% (*p*-value < 0.05) will be assumed.

The selected sample will include all patients diagnosed with colon cancer from the General Surgery Service of various public hospitals in the province who have surgical indications as part of their therapeutic plan over a recruitment period of 6 months and who voluntarily consent to participate in the study.

However, given the study’s design characteristics, a non-probabilistic sampling approach was adopted. Sample size calculation was performed using the Fisterra tool [45]. A sample of 70 patients, based on a population of 338 patients with abdominopelvic cancer who underwent surgical intervention in previous years in the hospitals of reference, would be sufficient to estimate a representative sample of the study population with a 95% confidence level and a precision of +/− 5 percentage points. A necessary replacement rate of 15% has been considered.

Ethical Considerations: The implementation of this project focuses on gathering information and assessing a prehabilitation program on self-care and targeted physical exercise in patients diagnosed with colon or gynecological cancer. It does not involve any intervention on the participant with any medical technique or invasive procedure that could affect them. Likewise, there will be no experimental component that could jeopardize the patient’s health, thus preserving the principle of non-maleficence.

The research will be conducted according to the Declaration of Helsinki, and it was approved by the Research Ethics Committee of the Jaume I University of Castellon (Expedient Number CEISH/87/2023) and registered at Clinical.Trials.gov (ID: NTC05997531), and we use a checklist for the drafting of a health research protocol to enhance the quality and transparency of the project [46].

All participants will be required to sign an informed consent form, which will include an invitation to participate in the study, the study’s objectives, a description of the procedures they will undergo, and the potential risks and benefits associated with participation. Additionally, participants will be provided with information regarding the data to be collected from their participation in the study, as well as methods to ensure anonymity and confidentiality. They will be given contact information for the project’s designated point of contact for resolving any doubts, and they will be informed of the procedure for withdrawing from the study.

## 3. Discussion

When a patient is diagnosed with cancer, multiple intense emotional reactions coexist, impacting not only their biological dimension but also their psychological, familial, social, and occupational dimensions [1,4,47]. The disruption in all these dimensions affects the quality of life of these patients [1,48] and leads to a decrease in their ability to self-care [47].

Historically, the term “self-care” was coined by Dorothea Orem in the 1950s. This nurse first defined the concept of self-care as “an activity that individuals learn and perform in specific life situations, aimed at the same goal, enabling the person to be independent and promote the necessary conditions to preserve life for the benefit of health and well-being” [47,49].

In her theoretical model, Orem delineates the requirements (activities) that individuals must undertake to meet their self-care objectives, distinguishing between universal self-care requisites, developmental self-care requisites, and self-care requisites in health deviation situations [49].

When a disease conditions a person, as is the case with a cancer diagnosis, it requires specific self-care measures [49]. At this point, empowerment becomes a relevant factor, involving assisting the patient in utilizing their self-care capabilities [49]. This is the only protocol in the literature that has considered the patient’s self-care capacity as part of its objectives.

Nursing care for patients before undergoing surgery focuses on the psychological needs of both the patient and their family, the physical condition of the patient, and educating the patient and their environment about the procedures they will undergo, among other aspects [48]. Kamarajah et al. [50], in their systematic review, mention the article by Kalogera et al. in which they reported a reduction of stress in patients who received nurse-led preoperative education in patients undergoing major cardiac surgery but mention that there is no evidence available for major abdominal surgery, which underlines the need for more research in this area, which our study includes health education and stress reduction through the intervention of a referring nurse.

In the realm of health complications stemming from cancer treatment, which often entail multifaceted deficiencies, the rationale for implementing a multimodal Prehabilitation becomes evident. This approach facilitates the comprehensive targeting of various aspects, spanning from physical to psychological interventions. By integrating a spectrum of strategies, prehabilitation offers a more holistic approach to treatment enhancement compared to singular interventions, ultimately enriching the overall patient experience [1,9]. However, the real benefits of prehabilitation remain a topic of debate, as the current evidence is very contradictory [51,52] and heterogenic [9,52]. Unfortunately, the evidence regarding multimodal prehabilitation programs before surgery is limited [3,9,10,15,53,54]. The modalities of prehabilitation programs are very diverse. Many programs remain unimodal and focus exclusively on physical training [55]. For example, in the meta-analysis carried out by Cambriel et al. [56], only 25% of the selected articles were studies in which a multimodal intervention was carried out.

Multimodal programs that include physical training, nutritional support, psychological support, and interaction between these components [50,55], such as our protocol, may be the most effective [55] and maximize the impact on postoperative outcomes [57] and should be considered in future research [55]. At present, results should be approached with caution given the significant variability within and between studies, the small sample size of the included studies, and the incomplete reporting of exercise interventions, which may limit the robustness of the results [50].

The optimal approach to delivering prehabilitation remains unknown, as programs vary in exercise type, training frequency, intensity, duration, and supervision, affecting their therapeutic validity [52]. Due to this, more research, such as our multicenter study, is necessary to verify the effects and to explore implementation strategies within clinical practice [52], which we have attempted to address with sample size calculation despite convenience sampling and give greater detail of what the physical exercise interventions are like.

Multimodal oncological prehabilitation offers an opportunity to support and prepare these patients for their future therapeutic plans and to enhance their self-care agency or capacity. The preoperative period offers an opportunity to prepare patients for the upcoming physiological and psychological stress [50]. Additionally, it provides the opportunity to transform a waiting period characterized by a passive role until surgery into an opportunity for empowerment and active optimization for these patients.

Besides, this study has the potential to address indirectly or directly several of the top 10 research priorities defined by the Delphi study, among which are the effect of prehabilitation on surgical outcomes, identifying populations most likely to benefit from prehabilitation, the optimal composition of prehabilitation programs, the effect of prehabilitation on patient-reported outcomes, modes of delivery for prehabilitation, optimal exercise modality and duration of prehabilitation programs, patient education, medical optimization, and psychological interventions [58].

## 4. Conclusions

In this context of health deviation and given the complex nature of the deficiencies associated with many cancer treatments, a multimodal prehabilitation is justified, allowing for the addressing of a wide spectrum of interventions, thereby enhancing the overall treatment experience better than any isolated intervention. However, studies related to Prehabilitation in colon or gynecological cancer are highly heterogeneous in terms of intervention and investigated outcomes, and further evidence is needed to determine the optimal Prehabilitation program for these patients. The analysis of selected variables and the use of validated tools will help to correct and elaborate upon care guidelines, providing better assistance to patients and their caregivers.

## Figures and Tables

**Table 1:** Primary variables.

Primary Variables			
Outcomes	Measurement	Outcomes	Measurement
Physical Variables		Fitness variables	
Anthropometry	Bioimpedance [22]	Functional capacity	Walking Test 6′ [23]
Blood Pressure	Tensiometer [24]	Upper limb strength	Hand Grip [25,26,27]
Cardiac frequency	Tensiometer [24]	Lower limb strength	Squat Jump andSit and up [28,29]
Oxygen saturation	Pulse-oximeter	Flexibility	Seat and Reach [30]
Psychological Variables			
Outcomes	Measurement	Outcomes	Measurement
Anxiety	State Trait Anxiety Inventory [31,32]	Depression	Hospital Anxiety and Depression Scale [33,34,35,36]
Self-care and health variables			
Outcomes		Measurement	
Appraisal of self-care		Appraisal of self-care agency scale [37,38]	
Lifestyle		Accelerometer sensor GENEActiv [30,31,32,33,34,35,36,37,38,39,40]	
Quality of life		EORTIC Quality of life questionnaires [41,42]	
Nutrition screening		Malnutrition Universal Screening Tool (MUST) [43]	
Fragility		FRAIL scale [44]	

Note: Variables included in the study.

## Data Availability

For ethical reasons related to the preservation of patient identity, the data presented in this study are available upon request to the corresponding author.
